# Virulence of *Candida auris* from different clinical origins in *Caenorhabditis elegans* and *Galleria mellonella* host models

**DOI:** 10.1080/21505594.2021.1908765

**Published:** 2021-04-12

**Authors:** Ainara Hernando-Ortiz, Estibaliz Mateo, Aitzol Perez-Rodriguez, Piet W.J. de Groot, Guillermo Quindós, Elena Eraso

**Affiliations:** a Department of Immunology, Microbiology and Parasitology, Faculty of Medicine and Nursery, University of the Basque Country (UPV/EHU), Bilbao, Spain; b Regional Center for Biomedical Research, Castilla-La Mancha Science & Technology Park, University of Castilla-La Mancha, Albacete, Spain

**Keywords:** *Candida auris*, emerging pathogen, candidiasis, virulence, invertebrate models

## Abstract

*Candida auris* is an emerging multidrug-resistant fungal pathogen responsible for nosocomial outbreaks of invasive candidiasis. Although several studies on the pathogenicity of this species have been reported, the knowledge on *C. auris* virulence is still limited. This study aims to analyze the pathogenicity of *C. auris*, using one aggregating isolate and eleven non-aggregating isolates from different clinical origins (blood, urine and oropharyngeal specimens) in two alternative host models of candidiasis: *Caenorhabditis elegans* and *Galleria mellonella*. Furthermore, possible associations between virulence, aggregation, biofilm-forming capacity, and clinical origin were assessed. The aggregating phenotype isolate was less virulent in both *in vivo* invertebrate infection models than non-aggregating isolates but showed higher capacity to form biofilms. Blood isolates were significantly more virulent than those isolated from urine and respiratory specimens in the *G. mellonella* model of candidiasis. We conclude that both models of candidiasis present pros and cons but prove useful to evaluate the virulence of *C. auris in vivo*. Both models also evidence the heterogeneity in virulence that this species can develop, which may be influenced by the aggregative phenotype and clinical origin.

## Introduction

*Candida albicans* is the predominant etiological agent of invasive candidiasis [1,2]. However, recently *Candida auris* is emerging as a major cause of invasive nosocomial infections with multidrug-resistance and high morbidity and mortality [3,4]. Since this species was first described in 2009, several clinical presentations, such as colonization, mucosal infection or bloodstream infection have been reported worldwide, mostly associated with outbreaks in surgical and medical intensive care units (ICU) [5,6]. Phenotypic and genotypic characterization studies of *C. auris* isolates from different countries have revealed the high diversity of this species identifying up to five possible different phylogeographical clades, four clearly distinct and a potential fifth clade, around the world [6,7].

A wide array of virulence mechanisms is encoded in the genome of *C. auris* [8]. Expression of phospholipase, proteinase, and hemolytic activities, adherence and biofilm formation, antifungal drug and environmental stress resistance are described among the most relevant virulence traits [9–11]. However, the knowledge on *C. auris* virulence remains scarce and further studies are required to understand its pathogenic behavior, which has been reported to be substantially variable among clades [12]. Currently, about 30 studies have incorporated *in vivo* models in the analysis of *C. auris* virulence, murine models being the most widely used [13–18]. However, ethical and economical issues for using these models of candidiasis are encouraging the use of alternative systems such as invertebrate models [19,20]. Several virulence studies of *C. auris* infections have been reported in invertebrate models using *Caenorhabditis elegans, Drosophila melanogaster, Danio rerio* or *Galleria mellonella* [11,21–29]. Moreover, non-mammalian hosts have been successfully implemented for studying host-pathogen interactions and invasive candidiasis caused by other *Candida* species, and to evaluate the effectiveness of new therapeutic approaches [11,30–33].

Advances in the knowledge of the virulence potential of *C. auris* will contribute to the control of infections by this emerging pathogen. Recently, two different phenotypes, aggregating and non-aggregating (single-cell phenotype), have been described among *C. auris* clinical isolates; the latter presenting a higher virulence [21,22,26]. Transcriptional profiles were evidently different between isolates with these two different phenotypes, and differences in the host response, depending on whether there is loss of tissue integrity, were also reported [12]. Although attempts have been made to correlate the site of origin of clinical isolates with their virulence in different *Candida* species [34–37], no association has been reported between the pathogenicity of *C. auris* and the origin of clinical specimens.

Therefore, the aim of this study was to analyze the virulence of *C. auris* isolates including both, aggregating and non-aggregating phenotypes that were retrieved from different clinical specimens. The ability of several *C. auris* isolates to form biofilms and produce hemolytic and enzymatic activity was assessed *in vitro* and the virulence traits of these isolates were probed *in vivo* using the invertebrate model hosts, *C. elegans* and *G. mellonella*. Our results demonstrate that both model organisms can be killed by aggregative as well as non-aggregative *C. auris* isolates. Moreover, this study reaffirmed that the variability among *C. auris* isolates as well as the site of infection and the different immune response of the host could affect this species virulence, being the *C. auris* blood isolates the most virulent.

## Materials and methods

### Candida auris *isolates and growth conditions*

Twelve clinical *C. auris* isolates from different patients suffering from candidiasis were analyzed (Table 1). These isolates included five from blood, five from urine, and two from oropharynx. All but one of the isolates came from patients of the Hospital Universitario y Politécnico La Fe of Valencia, Spain (Dr. Alba Ruiz Gaitán and Dr. Javier Pemán), three of them being registered in the CBS-KNAW culture collection of Westerdijk Fungal Biodiversity Institute. One blood isolate, *C. auris* JMRC:NRZ 1101 (Jena Microbial Resource Collection) was recovered from a patient attended at the Institut für Hygiene und Mikrobiologie, Würzburg, Germany (Dr. Oliver Kurzai).

**Table 1.** Biofilm biomass and metabolic activity levels of each *C. auris* isolate measured with Crystal violet and XTT reduction assays, respectively

			Biomass of biofilm		Metabolic activity of biofilm	
Species	Origin site	Isolate	OD (24 h)	OD (48 h)	OD (24 h)	OD (48 h)
*Candida albicans*		SC5314	0.719 ± 0.066	0.696 ± 0.071	1.159 ± 0.154	1.247 ± 0.172
*Candida auris*	blood	JMRC:NRZ 1101*	0.508 ± 0.047	0.361 ± 0.039	0.421 ± 0.147	0.357 ± 0.075
		CJ94	0.068 ± 0.038	0.080 ± 0.046	0.145 ± 0.070	0.122 ± 0.031
		CBS15605	0.074 ± 0.049	0.079 ± 0.048	0.156 ± 0.086	0.124 ± 0.033
		CBS15606	0.068 ± 0.042	0.083 ± 0.051	0.135 ± 0.062	0.103 ± 0.028
		CBS15607	0.086 ± 0.079	0.077 ± 0.043	0.132 ± 0.074	0.098 ± 0.023
	urine	CR14	0.227 ± 0.034	0.179 ± 0.054	0.237 ± 0.095	0.114 ± 0.060
		CR201	0.080 ± 0.036	0.057 ± 0.023	0.118 ± 0.035	0.122 ± 0.067
		CR220	0.103 ± 0.038	0.062 ± 0.030	0.119 ± 0.020	0.102 ± 0.048
		CR424	0.101 ± 0.024	0.082 ± 0.026	0.057 ± 0.039	0.056 ± 0.045
		CR440	0.090 ± 0.027	0.074 ± 0.025	0.121 ± 0.033	0.115 ± 0.055
	oropharyngeal	CR243	0.065 ± 0.033	0.084 ± 0.035	0.146 ± 0.056	0.119 ± 0.042
		CR312	0.081 ± 0.024	0.085 ± 0.046	0.085 ± 0.030	0.087 ± 0.047

OD: optical density

*The *C. auris* JMRC:NRZ 1101 blood isolate displayed an aggregating phenotype

*C. auris* isolates were stored in vials containing sterile distilled water at room temperature and cultured on Sabouraud dextrose agar (Difco, Becton Dickinson, USA) at 37°C for 24 h before use. The assays in the *C. elegans* model were performed by culturing *C. auris* on brain heart infusion (BHI, Panreac, Spain) agar plates supplemented with kanamycin (90 µg/ml) and incubated at 37°C for 24 h. *C. auris* isolates used for the *G. mellonella* model assay and for biofilm studies were cultured in yeast extract peptone dextrose broth (YEPD, Panreac) and incubated overnight at 30°C in shaking conditions.

### Microscopic visualization

The cellular morphology of the 12 clinical *C. auris* isolates was observed microscopically. *C. auris* isolates were grown in YEPD broth for 24 h at 30°C in shaking conditions, washed three times with sterile phosphate-buffered saline solution (PBS, Sigma-Aldrich, USA) and collected by centrifugation at 2500 rpm for 10 min. Cell suspensions of each *C. auris* isolate were adjusted to a final concentration of 1 × 10^8^ cells/ml with sterile PBS after cell counting by microscopy using a Burker hemocytometer. Microscopic appearance of each sample was visualized using a Nikon Eclipse 80i fluorescence microscope (Melville, NY, USA).

### Biofilm development

The ability of *C. auris* isolates to develop biofilms was assessed with *C. albicans* strain SC5314 being included as control. Biofilms were produced in sterile, flat-bottomed honeycomb 100-well polystyrene microtiter plates (Labsystems, Finland). Precultured cells were harvested and washed three times with sterile PBS. After cell counting by microscopy, suspensions at a concentration of 1 × 10^6^ cells/ml were prepared in RPMI 1640 medium supplemented with L-glutamine and buffered at pH 7 with 0.165 M 3-(N-morpholine) propanesulfonic acid, MOPS (Sigma-Aldrich). A volume of 100 µl of each cell suspension was dispensed into the wells of the plate. All outer wells from the first and last column of the plate were kept empty to avoid the “edge-effect”. Microplates were incubated at 37°C for 24 or 48 h. Afterward, to carefully eliminate planktonic and poorly adhered cells, spent RPMI was removed and the biofilms were washed three times by adding and removing 100 µl of sterile PBS.

Quantification of biofilm biomass was performed by Crystal violet (CV) staining [38]. After drying the washed biofilms at room temperature for 30 min, 100 µl of 0.4% CV solution (Merck, Germany) was added to each well and incubated for 20 min at room temperature. Each well was then washed twice with 250 µl of sterile distilled water, and finally 150 µl of 33% acetic acid was added to solubilize the CV-stained biomass for spectrophotometric measurement.

Biofilm metabolic activity was evaluated by reduction of 2,3-bis (2-methoxy- 4-nitro-5-sulfophenyl)-5-[(phenylamino)-carbonyl]- 2 H-tetrazolium hydroxide (XTT, Sigma-Aldrich) [39]. A volume of 100 µl of XTT with 1 μM of menadione was added to each washed well, and microplates were incubated in darkness for 2 h at 37°C.

Absorbance measurements were conducted with a BioScreen C MBR microplate reader (Growth Curves Ltd, Finland) at a wavelength of 600 nm for biomass quantification and at 492 nm for metabolic activity determination. Each experiment was performed with biological triplicates on separate days.

### Determination of phospholipase, proteinase and hemolytic activities

Phospholipase activity was determined using a precipitation assay as described by Polak (1992) [40] with malt agar plates supplemented with egg yolk [41]. Phospholipase activity was defined as the ratio of the colony diameter compared to the total diameter of colony plus precipitation zone. *C. albicans* isolate UPV/EHU 04–125 was used as a control with high phospholipase activity.

Aspartyl proteinase activity was assayed as described by Cassone et al. (1987) [42]. Proteinase activity was determined as the diameter of the lytic area around growing colonies. High aspartyl proteinase producer strain *Candida dubliniensis* UPV/EHU 00–134 was used as a control.

Hemolytic activity was analyzed as described by Luo et al. (2001) [43] by performing the plate assay described by Manns et al. (1994) [44]. *C. albicans* ATCC 90028 with high hemolytic activity was used as a control.

Enzymatic and hemolytic activities were analyzed at least with biological triplicates on separate days.

### *Survival assays in* Caenorhabditis elegans

*C. elegans* strain AU37 (*glp-4*(*bn2); sek-1*(*km4*)) was obtained from the Caenorhabditis Genetics Center (University of Minnesota, USA). The double mutation in this strain generates nematodes that are not able to reproduce at 25°C (*glp-4*) and are more susceptible to infection (*sek-1*). The nematodes were kept in the laboratory at 15°C in plates with nematode growth medium (NGM, 3 g of NaCl, 17 g of agar, 2.5 g of peptone, 1 ml of 1 M CaCl_2_, 1 ml of 5 mg/ml cholesterol in ethanol, 1 ml of 1 M MgSO_4_, 25 ml 1 M KPO_4_, 975 ml H_2_O) seeded with the nonpathogenic *Escherichia coli* strain OP50 as nourishment. Prior to survival experiments, nematodes were synchronized to the same growth stage, as described by Ortega-Riveros et al. (2017) [32]. Nematodes were then placed on BHI agar plates supplemented with kanamycin (90 µg/ml) and seeded with the isolate of *C. auris* to be assayed to feed the nematodes with the yeast [45]. After incubating the plates at 25°C for 2 h, the nematodes were washed with M9 buffer (3 g of KH_2_PO_4_, 6 g of Na_2_HPO_4_, 5 g of NaCl, 1 ml of 1 M Mg SO_4_ and H_2_O to 1 l) supplemented with kanamycin (90 µg/ml) and placed in plates with NGM agar for 15 min to eliminate, by friction, *C. auris* cells that might have adhered to the nematode cuticle. Nematodes were placed in groups of 20 individuals in 24-well plates containing M9 buffer supplemented with 10 μg/ml of cholesterol in ethanol and kanamycin (90 µg/ml). Nematode survival was monitored every 24 h until 120 h with a stereomicroscope (Nikon SMZ-745, Japan). A minimum of 60 nematodes were used in each experiment for each *C. auris* isolate, and groups of uninfected nematodes were included as controls. The experiments were carried out in triplicate on different days.

### *Survival tests in* Galleria mellonella

*G. mellonella* larvae with a weight between 0.3 and 0.5 g were obtained from Bichosa (Spain). Experiments were started one day after caterpillars arrived, which were placed in groups of 20 individuals in Petri dishes. The last left pro-leg of each larvae was cleaned with 70% ethanol and 10 µl of a *C. auris* suspension were inoculated with a precision syringe (Agilent, USA). Inoculum of *C. auris* cells was prepared by washing overnight yeast cultures with PBS supplemented with ampicillin (20 μg/ml) to remove remnants of YEPD and prevent bacterial contamination. Concentrations of 1 × 10^5^, 1 × 10^6^ and 1 × 10^7^ cells/larva were assayed to monitor the virulence of each *C. auris* isolate. In all trials, two uninfected groups were used as controls; one with untouched larvae and a sham group with larvae inoculated with 10 µl PBS-ampicillin to observe a possible effect of the injection. Survival counts of alive and dead larvae were determined by visual inspection of movement and melanization every 24 h until 120 h after infection. Trials were conducted in triplicate on three different days.

### Statistics

The quantitative results obtained in biofilm production were analyzed using the Student’s *t* test of the statistical program SPSS v24.0 (IBM, Chicago, IL, USA). The analysis of the virulence of *C. auris* isolates according to the origin of clinical specimens was analyzed by one-way ANOVA using SPSS v24.0. Survival curves were prepared with the Kaplan–Meier method using GraphPad Prism 5 software (GraphPad Software, La Jolla, CA, USA). Differences between the survival rates in both invertebrate models infected with *C. auris* were analyzed by the log-rank test of SPSS v24.0. For all statistical analyses values of p < 0.05 were considered statistically significant.

## Results and discussion

In the present study, we characterized virulence traits of 12 clinical isolates of *C. auris in vitro* and compared their capability to infect and kill *C. elegans* and *G. mellonella in vivo*. These *C. auris* isolates, that were recovered from patients at two different locations (Spain and Germany), were observed microscopically, and only the blood isolate JMRC:NRZ 1101 from Germany displayed an aggregating phenotype, which was not observed in the other *C. auris* isolates from Spain (Figure S1).

### *Biofilm formation, enzymatic activity, and hemolytic activity of* Candida auris

The ability of the 12 *C. auris* clinical isolates to develop biofilms is summarized in Table 1. None of the 12 isolates produced a biofilm denser than the *C. albicans* SC5314 control strain (Table 1). The ability to form a biofilm is an important factor in *Candida* pathogenicity because fungal cells within biofilms are protected from the action of the immune system and antifungal agents. The lower *C. auris* biofilm production compared to *C. albicans* SC5314 has been reported previously [9]. Blood isolate JMRC:NRZ 1101 with aggregating phenotype showed the highest biofilm biomass production and metabolic activity (p ≤ 0.0001). The non-aggregating urine isolate CR14 produced the second most dense biofilm with a significantly higher biomass (p ≤ 0.0001) and metabolic activity (p ≤ 0.02, except at 48 h for most of isolates) than the other *C. auris* isolates. Although *C. auris* biofilms are thinner than those of *C. albicans* [46], the capacity of *C. auris* to form a biofilm has been clearly associated with a lower antifungal susceptibility [16,47]. The ability of *C. auris* to form a biofilm has been reported for both aggregating and non-aggregating phenotypes [12]. Biomass values obtained in the current study were similar to those reported by Sherry et al. (2017) [22]. However, these authors reported the highest biofilm-forming capacity for non-aggregating isolates. Brown and coworkers [12] also detailed differences in gene expression between both phenotypes: genes related to cellular components (membrane and cell wall constituents) were upregulated in isolates with aggregating phenotype whereas genes related to biological processes and metabolic functions were expressed higher in non-aggregating isolates. Moreover, Short et al. (2019) [48] suggested that the ability of *C. auris* to form cellular aggregates increases survival of the yeast, which coincided with the upregulation of biofilm-associated genes. In addition, in *C. albicans*, the ability to produce biofilm has been associated with the expression of secreted aspartyl proteinases [49]. Genes involved in biofilm formation and in the production of phospholipase, proteinase and hemolysin activity have been reported in *C. auris*, with genes involved in cell adhesion and invasion (*ALS* and *SAP* families) showing stronger expression in aggregating than in non-aggregating isolates [8,12,13,50]. However, in the present study, phospholipase and proteinase activities were not detected in any clinical isolate nor any hemolytic activity was observed (Table S1).

### *Virulence of* Candida auris *in* Caenorhabditis elegans *and* Galleria mellonella

**Figure 1.**
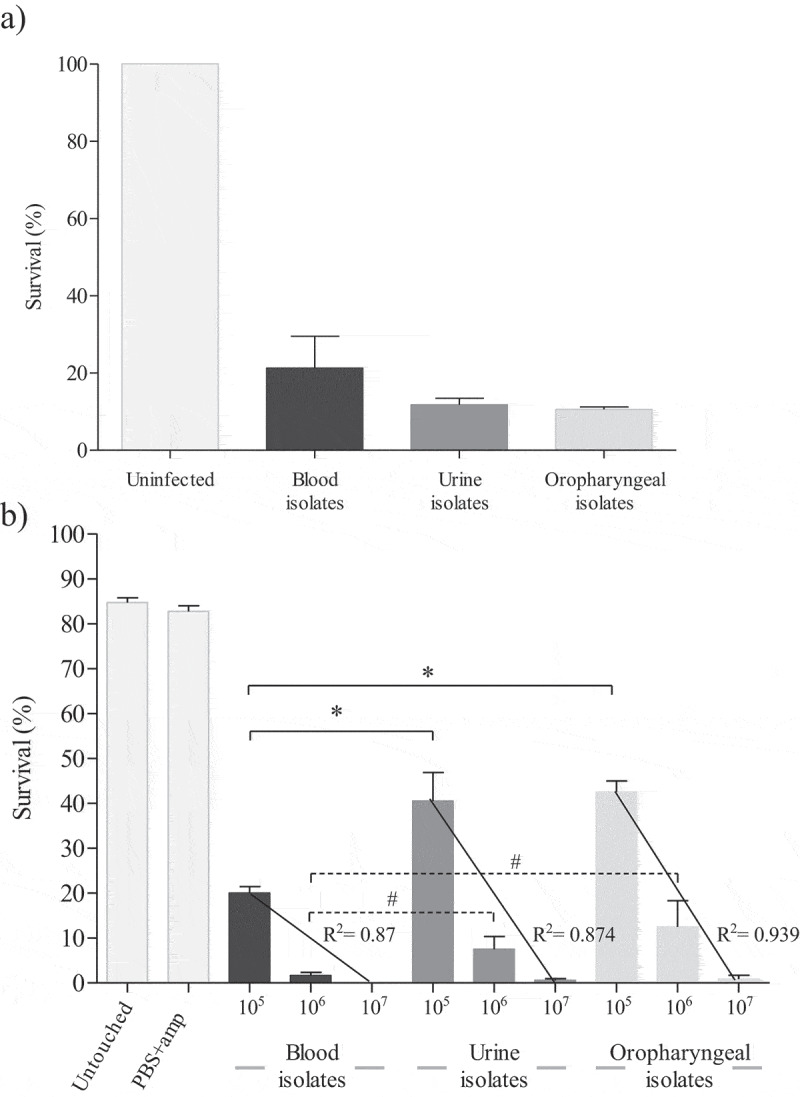
Survival average of *C. elegans* (a) and *G. mellonella* (b) at 120 hours post-infection with twelve different *C. auris* isolates according to the origin of the clinical specimen. Untouched control groups of nematodes and larvae and a group of larvae inoculated with PBS and ampicillin were also included. Larvae of *G. mellonella* were infected with three inocula of *C. auris* isolates (1 × 10^5^, 1 × 10^6^ and 1 × 10^7^ cells/larva) and correlation coefficients were calculated. Absent bars indicate 0% survival rate of *G. mellonella*. Error bars represent standard errors. Solid lines with asterisks denote statistically significant differences of the inoculum 1 × 10^5^ cells/larva between *C. auris* blood isolates and the other *C. auris* isolates from urine and oropharyngeal. Dotted lines with hashtag denote statistically significant differences of the inoculum 1 × 10^6^ cells/larva between *C. auris* blood isolates, without including the aggregative German isolate, and the other *C. auris* isolates from urine and oropharyngeal. The statistical analysis was performed using the one-way ANOVA test (p < 0.05)

*C. auris* virulence has been studied in murine [13–15,17,24], fish [28], and fly models [25]. However, there have only been a few studies in the nematode *C. elegans* [23,29] and the moth larvae *G. mellonella* [21,22,24,26,27]. In the present study, all *C. auris* isolates were able to cause the death of at least 47.7% of *C. elegans* and *G. mellonella* individuals after 120 h (Figure 1).

Uninfected *C. elegans* nematodes used as controls remained viable (100% survival) during the 120 h. A high amount of non-aggregating yeast cells was observed at 120 h post-infection for all *C. auris* isolates, except for the *C. auris* JMRC:NRZ 1101 isolate that caused yeast aggregates at 120 h post-infection in *C. elegans* (Figure S2). This cellular pattern was also observed in infected *G. mellonella* (data not shown). These results are consistent with those reported by Borman et al. (2016) [21] who first described the formation of aggregates by some *C. auris* isolates, which later was corroborated by Sherry et al. (2017) [22] and Muñoz et al. (2020) [11]. These studies reported that *C. auris* did not form hyphae unlike *C. albicans* in the *G. mellonella* model of candidiasis, and that non-aggregating *C. auris* isolates were more virulent, even when compared to some *C. albicans* isolates [11,21,22]. In a murine model, Ben-Ami et al. (2017) [13] also identified yeast cells during the infection and recovered aggregates from murine tissues. Yue et al. (2018) [15] observed a filamentous morphology of *C. auris* in cultures on YEPD medium from liver, kidney, brain, lung, and spleen specimens of mice suffering from invasive candidiasis. However, two days of incubation at 30 °C followed by five additional days at 25 °C were needed to observe this morphological switch from yeast to hyphae.

Killing assays in *G. mellonella* were performed with inocula of 1 × 10^5^, 1 × 10^6^ and 1 × 10^7^ cells/larva for each *C. auris* isolate. In both control groups, most larvae were alive by 120 h post-infection with survival averages of 85% ± 3.2% in the case of the untouched group control and 82.3% ± 3.6% in larvae inoculated with PBS-ampicillin (Figure 1). Survival of *G. mellonella* significantly decreased when a higher inoculum of *C. auris* was administered, as reported for other species of *Candida* [31,51,52]. There was a strong correlation (R^2^ ≥ 0.87) between the injected inocula of *C. auris* and the survival of *G. mellonella*. Interestingly, there were statistically significant differences between the virulence of *C. auris* blood isolates and that of urine and oropharyngeal isolates using the inoculum of 1 × 10^5^ cells/larva (p ≤ 0.009). Differences were also detected with the inoculum of 1 × 10^6^ cells/larva without considering the isolate with aggregating phenotype (p ≤ 0.032) (Figure 1). The inoculum of 1 × 10^6^ cells/larva appeared the most appropriate for analyzing the virulence of *C. auris* in the *G. mellonella* model based on the findings with all the *C. auris* isolates at the three different inocula and the mortality rates obtained. Therefore, the results obtained with 1 × 10^6^ cells/larva are presented and discussed in detail in the main text while those obtained with other inocula are presented in Figure S3. Inocula of 1 × 10^7^ cells/larva caused the death of more than 60% of the larvae during the first 24 h of infection, and of more than 98.3% at 120 h (Figure S3). On the opposite, with the lowest inoculum of 1 × 10^5^ cells/larva, none of the *C. auris* isolates had killed more than 60% of *G. mellonella* larvae at 48 h, with mortality ranging from 43.7% to 83.3% at 120 h (Figure S3). In this *in vivo* model the highest mortality was observed for blood isolates (Figure 1).

#### *Virulence of* C. auris *isolates according to the origin of the clinical specimens*

Differences in the expression of virulence factors have been observed between *C. auris* isolates from different geographical origins, such as the lack of adhesion or few biofilm production associated with clade II isolates (East Asian clade), which frequently cause otitis [12,53,54]. Several studies have associated the expression of specific virulence factors of *Candida* with the site of infection [34–37]. In the present study, *C. auris* isolates were not grouped within any specific clade but the eleven isolates from the Spanish outbreak are phylogenetically close to clade III isolates (South African clade) [5,6]. This clade III, as well as clade II, is associated with bloodstream infections and with aggregate formation [55].

We compared the virulence of the *C. auris* isolates according to their clinical origin in both *in vivo* models. The applied inoculum size of 1 × 10^6^ cells/larva or lower (2.5–5 × 10^5^ cells/larva) has also previously been used to establish the virulence of *C. auris* [21,22,24,26,27] and other species of *Candida* [30,51,52]. Strikingly, the *C. auris* isolates presented different virulence potential according to their clinical origin. In the *G. mellonella* model, the following general virulence categorization was observed when comparing the eleven non-aggregating isolates: blood > urine > oropharyngeal isolates with average survival percentages of infected larvae after 120 h of 1.7% for blood, 7.5% for urine, and 12.5% for oropharyngeal isolates. In contrast, mortality of *C. elegans* caused by infection with *C. auris* isolates was not clearly associated to their clinical origin. The average survival of nematodes infected with the non-aggregating *C. auris* isolates was very similar for the three groups of clinical isolates: 13.6% for the infection with blood isolates, 11.8% with urine isolates, and 10.6% with oropharyngeal isolates (Figure 1).

**Figure 2.**
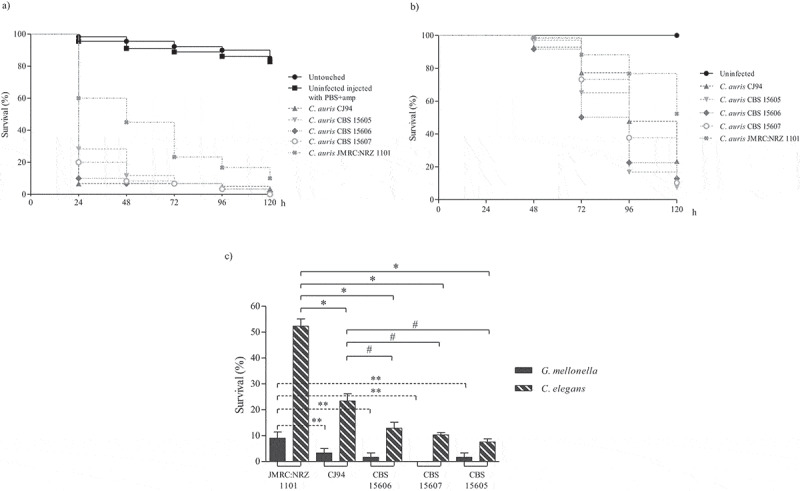
Survival curves of *G. mellonella* (a) and *C. elegans* (b) infected with *C. auris* blood isolates. Larvae of *G. mellonella* were infected with 1 × 10^6^ cells/larva and the control groups used were a group of untouched larvae and larvae inoculated with PBS and ampicillin (PBS+amp) as a puncture (sham) control group. *C. elegans* worms were infected by *C. auris* cell ingestion for 2 h. c) Survival percentages at 120 h post-infection of *G. mellonella* and *C. elegans* infected with *C. auris* blood isolates. The *C. auris* isolates were sorted from highest to lowest survival percentages of *C. elegans*. Statistically significant differences in pathogenicity of *C. auris* blood isolates compared to the least virulent isolate JMRC:NRZ 1101 (* *C. elegans*; ** *G. mellonella*) and the second least virulent isolate CJ94 (#) calculated using the log-rank test (p < 0.05) are indicated. Absent bars indicate 0% survival rate

**Figure 3.**
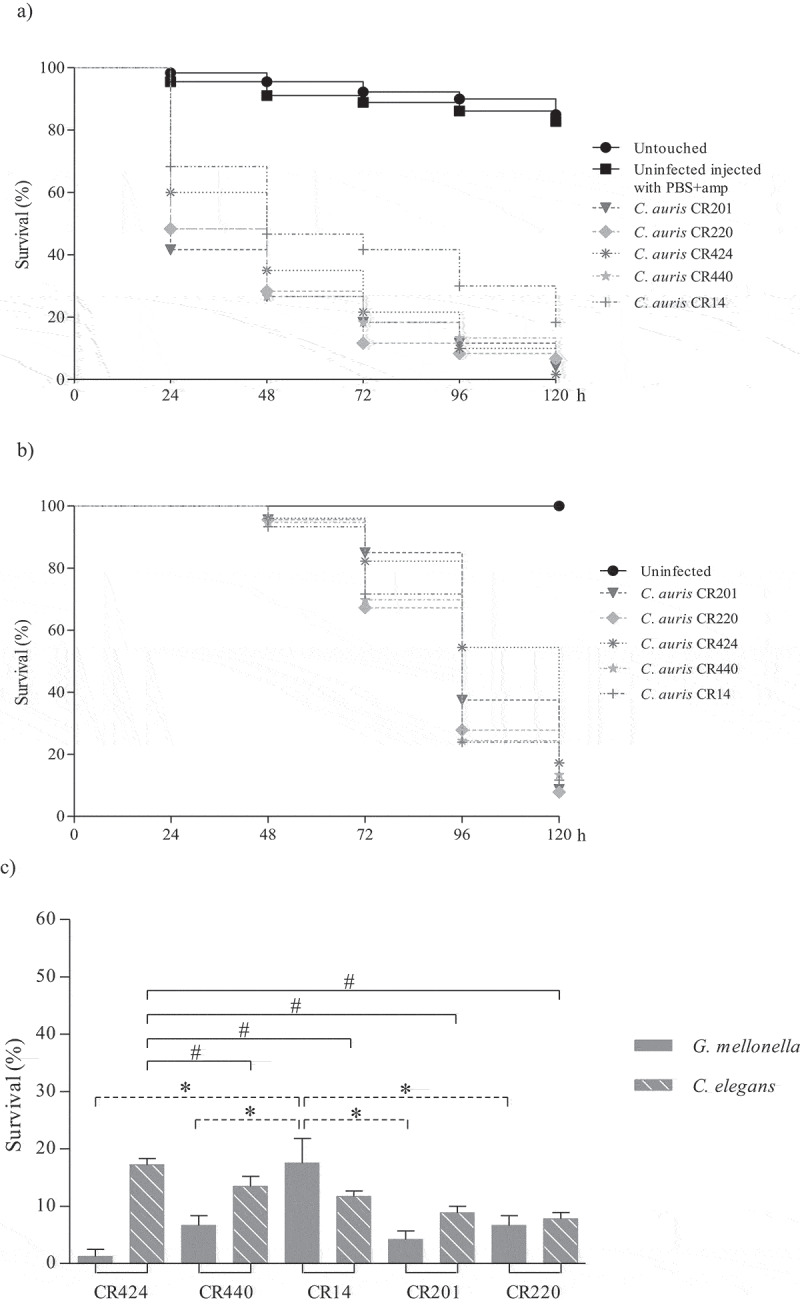
Survival curves of *G. mellonella* (a) and *C. elegans* (b) infected with *C. auris* urine isolates. Larvae of *G. mellonella* were infected with 1 × 10^6^ cells/larva and the control groups used were a group of untouched larvae and larvae inoculated with PBS and ampicillin (PBS+amp) as a puncture (sham) control group. *C. elegans* worms were infected by *C. auris* cell ingestion for 2 h. c) Survival percentages at 120 hours post-infection of *G. mellonella* and *C. elegans* infected with *C. auris* urine isolates. The *C. auris* isolates were sorted from highest to lowest survival percentages of *C. elegans*. Statistically significant differences in pathogenicity of *C. auris* urine isolates compared to the least virulent isolate in *G. mellonella, C. auris* CR14 (*), and the highest virulent isolate in *C. elegans, C. auris* CR424 (#), calculated using the log-rank test (p < 0.05) are indicated

**Figure 4.**
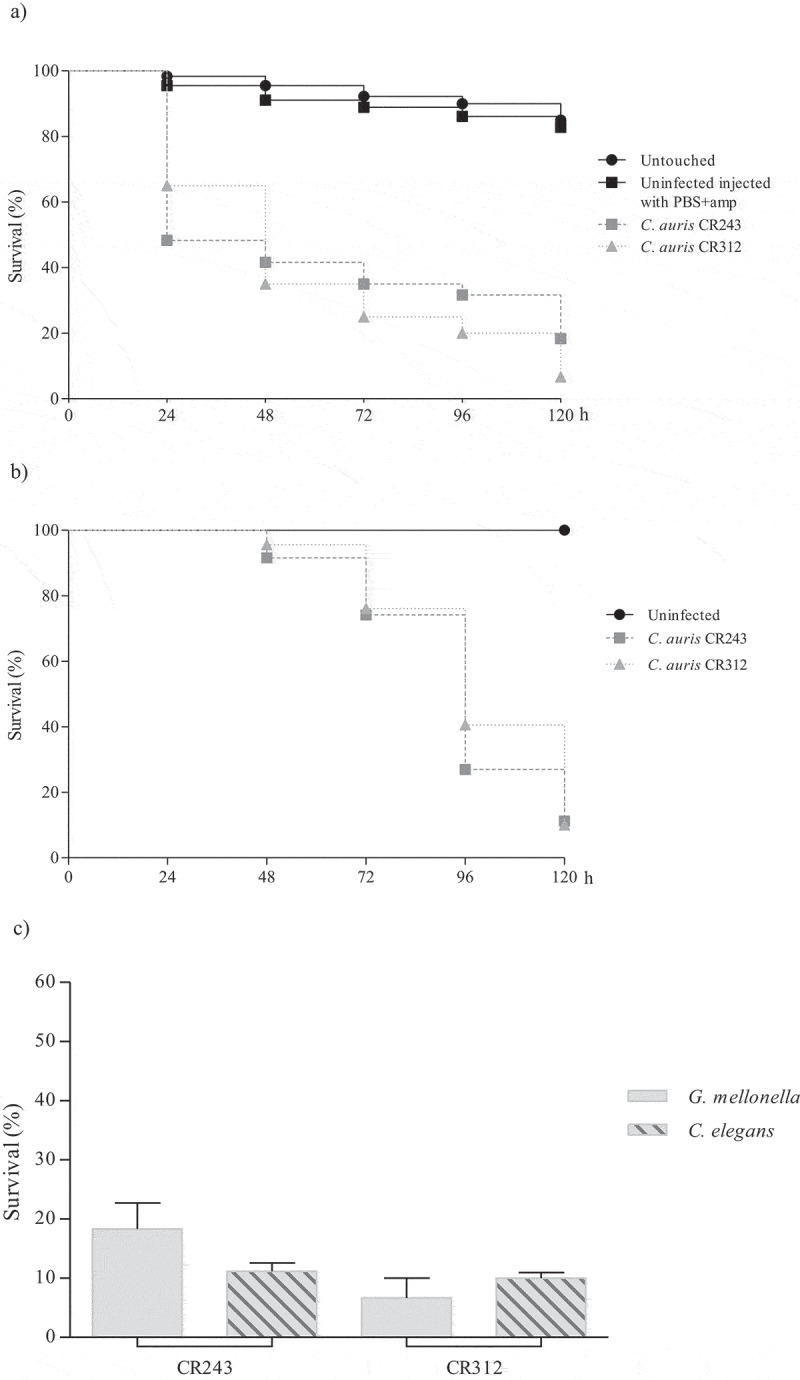
Survival curves of *G. mellonella* (a) and *C. elegans* (b) infected with *C. auris* oropharyngeal isolates. Larvae of *G. mellonella* were infected with 1 × 10^6^ cells/larva and the control groups used were a group of untouched larvae and larvae inoculated with PBS and ampicillin (PBS+amp) as a puncture (sham) control group. *C. elegans* worms were infected by *C. auris* cell ingestion for 2 h. c) Survival percentages at 120 hours post-infection of *G. mellonella* and *C. elegans* infected with *C. auris* oropharyngeal isolates. The *C. auris* isolates were sorted from highest to lowest survival percentages of *C. elegans.*

The virulence of the five individual blood isolates, the five urine isolates and the two oropharyngeal isolates is presented in Figures 2, 3 and 4, respectively. The survival rates of *G. mellonella* infected with blood isolates ranged from 0% to 10% at 120 h, and from 7.6% to 52.3% in the *C. elegans* model. Moreover, most of the *C. auris* isolates killed more than 70% of the *G. mellonella* larvae at 24 h but took more than 96 h in the *C. elegans* model (Figure 2). Survival rates for *G. mellonella* infected with urine isolates were between 1.7% and 18.3% at 120 h (7.8–17.2% in *C. elegans*). However, all urine isolates killed more than 50% of the *G. mellonella* larvae at 48 h, while in the case of *C. elegans* at least 96 h were required (Figure 3). Finally, for the two oropharyngeal isolates there were no significant differences in virulence between the two non-mammalian models: survival rates of *G. mellonella* at 120 h ranged from 6.7% to 18.3%, whereas those of *C. elegans* were between 10% and 11.2%. Once again, in *G. mellonella* both isolates killed more than 60% of the larvae after 48 h but in *C. elegans* they took 96 h to eliminate the same percentage of host organisms (Figure 4). In all cases, in the first 24–48 h post-infection *C. auris* caused higher mortality in the *G. mellonella* model than in the *C. elegans* model (Figures 2–4). These results are comparable to those obtained in other studies, which observed that *C. auris* caused death of *G. mellonella* within 48 h [21,22] and of *C. elegans* between 48 and 96 h post-infection [23,29]. Our findings corroborate the idea that both models successfully demonstrate the virulence potential of *C. auris*, which was similar to or higher than that of other *Candida* species [11,30,32,33,52]. The observed virulence of *C. auris* was comparable to *C. albicans* [21,22,29] and higher than isolates from *C. haemulonii* complex species [11,29]. However, also within the species *C. auris* differences in virulence between isolates from different clades have been noted, probably associated with their genomic diversity [12].

The *C. auris* JMRC:NRZ 1101 blood isolate, with aggregating phenotype, was significantly less virulent than the other blood isolates in both models (p ≤ 0.0001) (Table S2 and S3). This isolate killed more than 80% of the *G. mellonella* larvae in 96 h, but it took 120 h to achieve a mortality rate of 52.3% of *C. elegans* nematodes (Figure 2). Association of an aggregative phenotype of *C. auris* with lower pathogenicity compared to non-aggregating counterparts has also already been described earlier [21,22]. Furthermore, the virulence of this aggregating isolate in *C. elegans* was also significantly lower than that of all non-aggregating isolates from different infection sites (p ≤ 0.0001) (Figures 3 and 4; Table S3). In *G. mellonella*, the aggregating isolate caused a mortality comparable to that of most isolates from other clinical origins (Figure 2 and Table S2) and only the mortality caused by this isolate was significantly lower than of urine isolate CR201 (p = 0.016).

Regarding non-aggregating *C. auris* isolates, blood isolate CBS15607 killed 100% of *G. mellonella* at 120 h but no significant differences were observed with other non-aggregating blood isolates (Figure 2). High virulence was observed for four out of five urine isolates (80%), which killed *G. mellonella* with a mortality rate ranging from 80% to 93.3% between 72 and 120 h without significant differences among them (Figure 3). The CR312 oropharyngeal isolate also killed 93.3% of larvae at 120 h (Figure 4); however, its killing kinetics were similar to the two isolates with the lowest virulence, urine isolate CR14 and oropharyngeal isolate CR243, which achieved 81.7% mortality at 120 h. Conversely, there were significant differences between the latter two isolates and the aforementioned four urine isolates (p ≤ 0.006 and p ≤ 0.045, respectively). A higher virulence of *Candida* isolates from blood specimens has previously been reported [35–37]. *C. elegans* infection by non-aggregating isolates caused a mortality rate of more than 76.6% at 120 h and *C. auris* isolates took more than 72 h to exceed a mortality of 50% (Figures 2–4). Eldesouky et al. (2018) [23] employed 30 min in the infection of worms and reported similar killing kinetics, whereas Lima et al. (2020) [29] infected *C. elegans* for four hours and detected a mortality rate of more than 70% at 72 h. Blood isolate CBS15605 and urine isolate CR220 killed the highest percentage of nematodes at 120 h, 92.7% and 92.2%, respectively. However, no significant virulence differences were observed among them and the CBS15606 blood isolate and two urine isolates (CR440 and CR14) (Table S3). Five out of eleven isolates (45.5%) showed the same killing kinetics. Moreover, the oropharyngeal isolates also showed high lethality, causing a mortality rate of 88.8% at 120 h without significant differences with the mortality caused by the CBS15607 blood isolate. Strikingly, the less virulent isolates in the *C. elegans* model were the CJ94 blood isolate and the CR424 urine isolate with similar killing kinetics, both being significantly different to the rest of the isolates (Table S3). However, both isolates were among the five most virulent against *G. mellonella*, and their mortality rates in the *C. elegans* and *G. mellonella* models differed: 82.8% versus 98.3% for the blood isolate and 76.6% versus 96.7% for the urine isolate, respectively.

It is noteworthy that non-aggregating isolates were more pathogenic than the aggregating isolate and that the aggregating isolate used in this study formed more biofilm than the non-aggregating isolates. Sherry et al. (2017) [22] reported the opposite, a higher biofilm formation capacity in non-aggregating than in aggregating isolates. The aggregating *C. auris* JMRC:NRZ 1101 blood isolate could be expressing genes implicated in biofilm formation and also genes involved with other virulence factors. The expression of these virulence genes could render a virulence of this isolate similar to that of some non-aggregating isolates, yielding similar killing kinetics during infection of *G. mellonella*. Transcriptional analysis of *C. auris* isolates revealed a high number of upregulated genes involved in biofilm formation and other pathogenic traits, which can be associated with the high resistance of this species to antifungal drugs [10,12]. In addition, differences in the infection profile and the host response to infection were observed for aggregating and non-aggregating phenotypes using a three-dimensional skin epithelial model. Although both phenotypes were more virulent in the presence of a wound, an aggregating isolate was more cytotoxic and proinflammatory than a non-aggregating one with more ability to evade the immune system [12]. Trying to explain the differences found between both models of candidiasis, it is important to mention that in *G. mellonella C. auris* inoculum was injected parenterally, achieving higher concentrations in the tissues. Moreover, a higher lethal capacity of the non-aggregating isolates versus the aggregating isolate was noted in this model, likely due to the differential immune response of the host. Arias et al. (2020) [26] suggested that, due to a higher ability to move and disseminate within *G. mellonella* tissues, yeast cells might be more virulent than aggregates of cells, with which we concur. In addition, *C. elegans* infection occurs by yeast ingestion during a defined time. Variations in exposure time consequently might result in a higher or lower fungal burden, leading to variations in the killing kinetics [32]. It may also be possible that *C. elegans* has greater difficulty in ingesting cellular aggregates than individual yeasts. This fact could lead to a lower intake of fungal burden resulting in a milder infection rather than an aggregating isolate being less virulent. A stronger cytokine response and lower macrophage lysis capacity and neutrophil recruitment have been reported for *C. auris* compared to *C. albicans* [18,28]. Interestingly, structurally unique *C. auris* mannoproteins contribute to a strong innate host defense in all five *C. auris* clades, although clade-specific differences were observed, clade V being the least immunogenic [28]. Both infection models, *C. elegans* and *G. mellonella*, present mechanisms and responses of the innate immune system that are conserved in mammals and may contribute to a better understanding of host-*C. auris* interactions.

Overall, in both models, isolates of *C. auris* behaved differently depending on their clinical origin, although the response to *C. auris* infection of the two models of candidiasis was also different. These differences in the virulence of *C. auris* according to the site of infection seem consistent with reports about the phenotypic plasticity of this species of *Candida* in response to environmental conditions, such as the passage through a mammalian body and variations in temperature to colonize a specific niche [15]. However, further studies are required using other *C. auris* isolates with aggregating phenotype, if possible, from different geographic clades, to assess more accurately their virulence *in vivo*. There may be a level of heterogeneity in virulence among aggregating *C. auris* isolates similar to that observed for non-aggregating isolates in this and other studies [12,18].

In conclusion, we demonstrated that both *C. elegans* and *G. mellonella* models of candidiasis are simple and clearly appropriate to assess the virulence of *C. auris* isolates. Likewise, these models are useful to detect variations in the virulence of clinical isolates with different origin and/or capacity to form cell aggregates. The model host *G. mellonella*, which allows a more precise and direct inoculation of the pathogen into the host tissues, revealed a significantly higher virulence for *C. auris* isolates from blood specimens. Among the successful strategies of *C. auris* to cause infection are its ability to evade neutrophil attack and to resist treatments with commonly used antifungal drugs. Therefore, obtaining a more effective and accurate therapy is one of the main targets on which to focus actions against this pathogen. These low-cost and manageable *in vivo* models are promising tools to analyze host-pathogen interactions or the effectiveness of current and new antifungal drugs against *C. auris*.

## Supplementary Material

Supplemental Material
