# Supplementary material for: Virulence of Candida auris from different clinical origins in Caenorhabditis elegans and Galleria mellonella host models
Source: Virulence. 2021 Apr 12;12(1):1063–75. doi: 10.1080/21505594.2021.1908765 (PMC8043173; doi:10.1080/21505594.2021.1908765)
Supplement: Supplemental Material [file KVIR_A_1908765_SM8534.docx]

**Supplementary data**

**Figure S1.** Visual appearance observed by microscopy of the twelve clinical *C. auris* isolates in PBS suspensions from a 24 h culture (20 × magnification).

|  |  |  |  |
| --- | --- | --- | --- |
| *Candida auris* JMRC:NRZ 1101 | *Candida auris* CJ94 | *Candida auris* CBS15605 | *Candida auris* CBS15606 |
|  |  |  |  |
| *Candida auris* CBS15607 | *Candida auris* CR14 | *Candida auris* CR201 | *Candida auris* CR220 |
|  |  |  |  |
| *Candida auris* CR424 | *Candida auris* CR440 | *Candida auris* CR243 | *Candida auris* CR312 |
|  |  |  |  |

**Figure S2.** Appearance of the non-aggregating phenotype (*C. auris* CBS15607) and aggregating phenotype (*C. auris* JMRC:NRZ 1101) at 120 h post-infection in *C. elegans*. Images were obtained with a stereomicroscope. Arrows point *C. auris* cell masses. The aggregates formed by isolate JMRC:NRZ 1101 are clearly different from the nebulous cell masses of the non-aggregating isolate CBS15607 (Scale bar=1000 µm).

| *Candida auris* CBS15607 |  | *Candida auris* JMRC:NRZ 1101 |
| --- | --- | --- |
|  |  |  |

**Figure S3.** Survival curves and survival percentages at 120 hours post-infection of *G. mellonella* infected with 1 × 10^5^ cells/larva (a) and 1 × 10^7^ cells/larva (b) of *C. auris* isolated from blood, urine and oropharyngeal specimens. Untouched and *G. mellonella* larvae inoculated with PBS and ampicillin (PBS+amp) were used as control groups. Statistically significant differences in pathogenicity between *C. auris* isolates compared to blood isolates *C. auris* CJ94 (*) and to the other three non-aggregating blood isolates (#) as determined using the log-rank test (p<0.05) are indicated. Absent bars indicate 0% survival rate.

- **1 × 10^5^ cells/larva of *Candida auris***

Survival curve of *Galleria mellonella* infected with *Candida auris* blood isolates

Survival curve of *Galleria mellonella* infected with *Candida auris* urine isolates

Survival curve of *Galleria mellonella* infected with *Candida auris* oropharyngeal isolates

Survival percentages of *Galleria mellonella* at 120 hours post-infection and statistically significant differences in pathogenicity between *C. auris* isolates

- **1 × 10^7^ cells/larva of *Candida auris***

Survival curve of *Galleria mellonella* infected with *Candida auris* blood isolates

Survival curve of *Galleria mellonella* infected with *Candida auris* urine isolates

Survival curve of *Galleria mellonella* infected with *Candida auris* oropharyngeal isolates

Survival percentages of *Galleria mellonella* at 120 hours post-infection

**Table S1.** Phospholipase, aspartyl proteinase, and hemolytic activities of *C. auris* isolates obtained from blood, urine and oropharyngeal specimens.

|  |  | Phospholipase activity | | |  | Aspartyl proteinase activity | |  | Hemolytic activity | | | |
| --- | --- | --- | --- | --- | --- | --- | --- | --- | --- | --- | --- | --- |
| *Candida* isolates |  | **Colony diameter (mm) ^c^** | **Halo diameter (mm)** | **Pz^b^** |  | **Halo diameter (mm)** | **Production** |  | **Colony diameter (mm)^c^** | **Halo diameter (mm)** | **Hz^b^** | **Hemolysis type** |
| *Candida* control strains^a^ |  | 18 | 34 | 0.53 |  | 1 | High |  | 10 | 15.3 | 1.53 | Beta  (complete hemolysis) |
| Blood isolates of *Candida* *auris* |  | 9-11 | Absence  of halo | - |  | Absence  of halo | - |  | 9-11 | Absence  of halo | - | Gamma  (no hemolysis) |
| Urine isolates of *Candida* *auris* |  | 9-11 | Absence  of halo | - |  | Absence  of halo | - |  | 9-11 | Absence  of halo | - | Gamma  (no hemolysis) |
| Oropharynx isolates of *Candida* *auris* |  | 9-11 | Absence  of halo | - |  | Absence  of halo | - |  | 9-11 | Absence  of halo | - | Gamma  (no hemolysis) |

^a^ *Candida albicans* UPV/EHU 04-125, *Candida dubliniensis* UPV/EHU 00-134 and *Candida albicans* ATCC 90028 were used as a control for phospholipase, aspartyl proteinase and hemolytic activities, respectively.

^b^ Phospholipase activity (Pz) and hemolytic activity (Hz) were calculated as the ratio between the diameter of the colony and the diameter of the halo in millimeters [41, 43].

^c^ Colony diameters of the different *C. auris* isolates are displayed in an interval.

**Table S2.** Statistical analysis of differences in pathogenicity between *C. auris* in *G. mellonella* infected with 1 × 10^6^ cells/larva.

| **Specimens** | Blood | | | |  | Oropharingeal | |  | Urine | | | | |  | Blood |
| --- | --- | --- | --- | --- | --- | --- | --- | --- | --- | --- | --- | --- | --- | --- | --- |
| ***C. auris* isolate** | CJ94 | CBS  150605 | CBS  150606 | CBS  150607 |  | CR243 | CR312 |  | CR201 | CR220 | CR424 | CR440 | CR14 |  | JMRC:NRZ 1101 |
| CJ94 |  |  |  |  |  |  |  |  |  |  |  |  |  |  |  |
| CBS 150605 | ns |  |  |  |  |  |  |  |  |  |  |  |  |  |  |
| CBS 150606 | ns | ns |  |  |  |  |  |  |  |  |  |  |  |  |  |
| CBS 150607 | ns | ns | ns |  |  |  |  |  |  |  |  |  |  |  |  |
| CR243 | 0.0001 | 0.0001 | 0.0001 | 0.0001 |  |  |  |  |  |  |  |  |  |  |  |
| CR312 | 0.0001 | 0.0001 | 0.0001 | 0.0001 |  | ns |  |  |  |  |  |  |  |  |  |
| CR201 | 0.002 | 0.019 | 0.001 | 0.002 |  | 0.003 | ns |  |  |  |  |  |  |  |  |
| CR220 | 0.002 | 0.021 | 0.001 | 0.002 |  | 0.026 | ns |  | ns |  |  |  |  |  |  |
| CR424 | 0.0001 | 0.002 | 0.0001 | 0.0001 |  | 0.025 | ns |  | ns | ns |  |  |  |  |  |
| CR440 | 0.001 | 0.012 | 0.001 | 0.001 |  | 0.045 | ns |  | ns | ns | ns |  |  |  |  |
| CR14 | 0.0001 | 0.0001 | 0.0001 | 0.0001 |  | ns | ns |  | 0.0001 | 0.002 | 0.004 | 0.006 |  |  |  |
| JMRC:NRZ 1101 | 0.0001 | 0.0001 | 0.001 | 0.0001 |  | ns | ns |  | 0.016 | ns | ns | ns | ns |  |  |

Virulence differences for all *C. auris* isolate combinations were analyzed using the log-rank test, and values of p<0.05 were considered statistically significant.

ns: not statistically significant differences (p>0.05).

**Table S3.** Statistical analysis of differences in pathogenicity between *C. auris* isolates in *C. elegans* infected by *C. auris* cell ingestion for 2 hours.

| **Specimens** | Blood | | | |  | Oropharingeal | |  | Urine | | | | |  | Blood |
| --- | --- | --- | --- | --- | --- | --- | --- | --- | --- | --- | --- | --- | --- | --- | --- |
| ***C. auris* isolate** | CJ94 | CBS  150605 | CBS  150606 | CBS  150607 |  | CR243 | CR312 |  | CR201 | CR220 | CR424 | CR440 | CR14 |  | JMRC:NRZ 1101 |
| CJ94 |  |  |  |  |  |  |  |  |  |  |  |  |  |  |  |
| CBS 150605 | 0.0001 |  |  |  |  |  |  |  |  |  |  |  |  |  |  |
| CBS 150606 | 0.0001 | ns |  |  |  |  |  |  |  |  |  |  |  |  |  |
| CBS 150607 | 0.0001 | 0.0001 | 0.0001 |  |  |  |  |  |  |  |  |  |  |  |  |
| CR243 | 0.0001 | 0.030 | 0.037 | ns |  |  |  |  |  |  |  |  |  |  |  |
| CR312 | 0.007 | 0.0001 | 0.001 | ns |  | ns |  |  |  |  |  |  |  |  |  |
| CR201 | 0.013 | 0.0001 | 0.0001 | ns |  | ns | ns |  |  |  |  |  |  |  |  |
| CR220 | 0.0001 | ns | ns | 0.020 |  | ns | 0.023 |  | 0.011 |  |  |  |  |  |  |
| CR424 | ns | 0.0001 | 0.0001 | 0.0001 |  | 0.0001 | 0.006 |  | 0.007 | 0.0001 |  |  |  |  |  |
| CR440 | 0.0001 | ns | ns | ns |  | ns | ns |  | ns | ns | 0.0001 |  |  |  |  |
| CR14 | 0.0001 | ns | ns | ns |  | ns | ns |  | 0.043 | ns | 0.0001 | ns |  |  |  |
| JMRC:NRZ 1101 | 0.0001 | 0.0001 | 0.0001 | 0.0001 |  | 0.0001 | 0.0001 |  | 0.0001 | 0.0001 | 0.0001 | 0.0001 | 0.0001 |  |  |

Virulence differences for all *C. auris* isolate combinations were analyzed using the log-rank test, and values of p<0.05 were considered statistically significant.

ns: not statistically significant differences (p>0.05).
